# Controlling beam trajectory and transport in a tapered helical undulator

**DOI:** 10.1107/S1600577524012463

**Published:** 2025-02-12

**Authors:** A. Fisher, J. Jin, P. Musumeci

**Affiliations:** a Department of Physics and Astronomy University of California Los Angeles 475 Portola Plaza Los Angeles CA90095 USA; SLAC National Accelerator Laboratory, USA

**Keywords:** free-electron lasers, pulsed wire, magnetic measurements, undulator tuning

## Abstract

In this paper we present a detailed discussion of the helical undulator system developed for high extraction efficiency experiments in the tapered-enhanced stimulated superradiant amplification regime. The design is based on permanent magnet technology and comprises two Halbach arrays orthogonally oriented and shifted by 90° with respect to each other. When used in low energy beamlines for THz generation, the electron beam trajectory and transport are particularly sensitive to the undulator off-axis fields so that it becomes important to complement on-axis field measurements with analysis and tuning of the higher-order field components. Here we describe how the pulsed wire measurement technique can be effectively used to retrieve on- and off-axis magnetic field characteristics of the undulator. A simple two-dipole model is developed to guide the final adjustments to the permanent magnet positions along the array to tune the quadrupole and sextupole components of the field.

## Abstract 2

In this work the transport of low energy electrons through helical magnetic undulators is discussed with application for THz generation in waveguide free-electron lasers. To optimize beam transmission, the off-axis fields sampled by low energy beams are tuned with a 3D pulsed-wire measurement technique that reveals localized errors in the quadrupole and sextupole field moments.

## 1. Introduction

Helical undulators can be used to maximize the energy exchange in free-electron laser (FEL) interactions since the coupling between the electromagnetic field and the electron beam is constant along the helical particle trajectory. Compared with planar undulators, where the coupling vanishes at the turning points in the sinusoidal trajectory, the FEL coupling can be increased by a factor or two or larger depending on the field strength (Palmer, 1972 ▸; Colson, 1977 ▸). At the same time, important technical shortcomings have limited the widespread use of helical undulators in FEL systems, with most projects defaulting to the simpler planar undulator geometry especially when radiation polarization is not a concern. First, helical undulators typically have a more closed geometry which limits access to the beam axis and creates engineering challenges for magnetic field measurements, beam diagnostics, and vacuum pumping. More importantly, tuning the radiation wavelength by adjusting the magnet gap is straightforward in planar systems by changing the mechanical separation between the two strongback jaws, but is at the very least cumbersome to implement for a helical geometry. Still, it is good to remember that the first FEL demonstration at Stanford by Madey *et al.* was performed with a helical bifilar coil (Elias *et al.*, 1976 ▸) and advanced designs for future X-ray free-electron lasers (XFELs) consider helical geometry interactions (Emma *et al.*, 2016 ▸; Balal *et al.*, 2023 ▸).

In order to increase the FEL energy extraction efficiency, the magnetic field amplitude (and possibly the undulator period) should be tapered along the interaction (Kroll *et al.*, 1981 ▸). In the last decade, University of California Los Angeles (UCLA) and RadiaBeam Technologies have developed tapered helical undulator technology for high efficiency interactions between electron beams and electromagnetic fields (Duris *et al.*, 2014 ▸; Sudar *et al.*, 2016 ▸; Duris *et al.*, 2015 ▸) culminating in the development of the Theseus undulator system (Park *et al.*, 2022 ▸). The design is based on permanent magnet Halbach arrays (Halbach, 1983 ▸) held together by four strongbacks with machined slots so magnets can be moved perpendicular to the beam axis with tuning screws, enabling tapering and tuning of the field amplitude and the particle trajectories.

One of the Theseus undulators has already been used in the Tessatron THz waveguide FEL at UCLA where 10% extraction efficiency was demonstrated from a 5.5 MeV electron beam in the 1 m undulator (Fisher *et al.*, 2022 ▸). Zero-slippage operation in the waveguide allows the radiation envelope to remain temporally aligned with a strongly compressed (sub-wavelength scale) electron beam enabling sustained interaction with strong seeding. However, in this regime the relatively low energy causes the electrons to wiggle with a beam trajectory amplitude which is roughly half of the waveguide radius, experiencing large focusing forces and strong coupling between the transverse coordinates. This poses a significant challenge in controlling the beam trajectory and transport including full transmission of the injected beam charge through the small aperture waveguide.

In this paper we study in detail the optical characteristics of helical undulators for low energy beams and develop a strategy for measuring and tuning the good field region of these devices on the measurement bench before installation. Traditional on-axis Hall probe or pulsed-wire scans are not sufficient to ensure correct tuning of the off-axis fields in the closed geometry. In order to optimize the beam trajectory and ensure maximal charge transmission through the waveguide, we utilize a pulsed-wiring setup and leverage the ability to translate the wire transversely with respect to the magnetic axis to sample and correct the off-axis fields.

**Figure 1:**
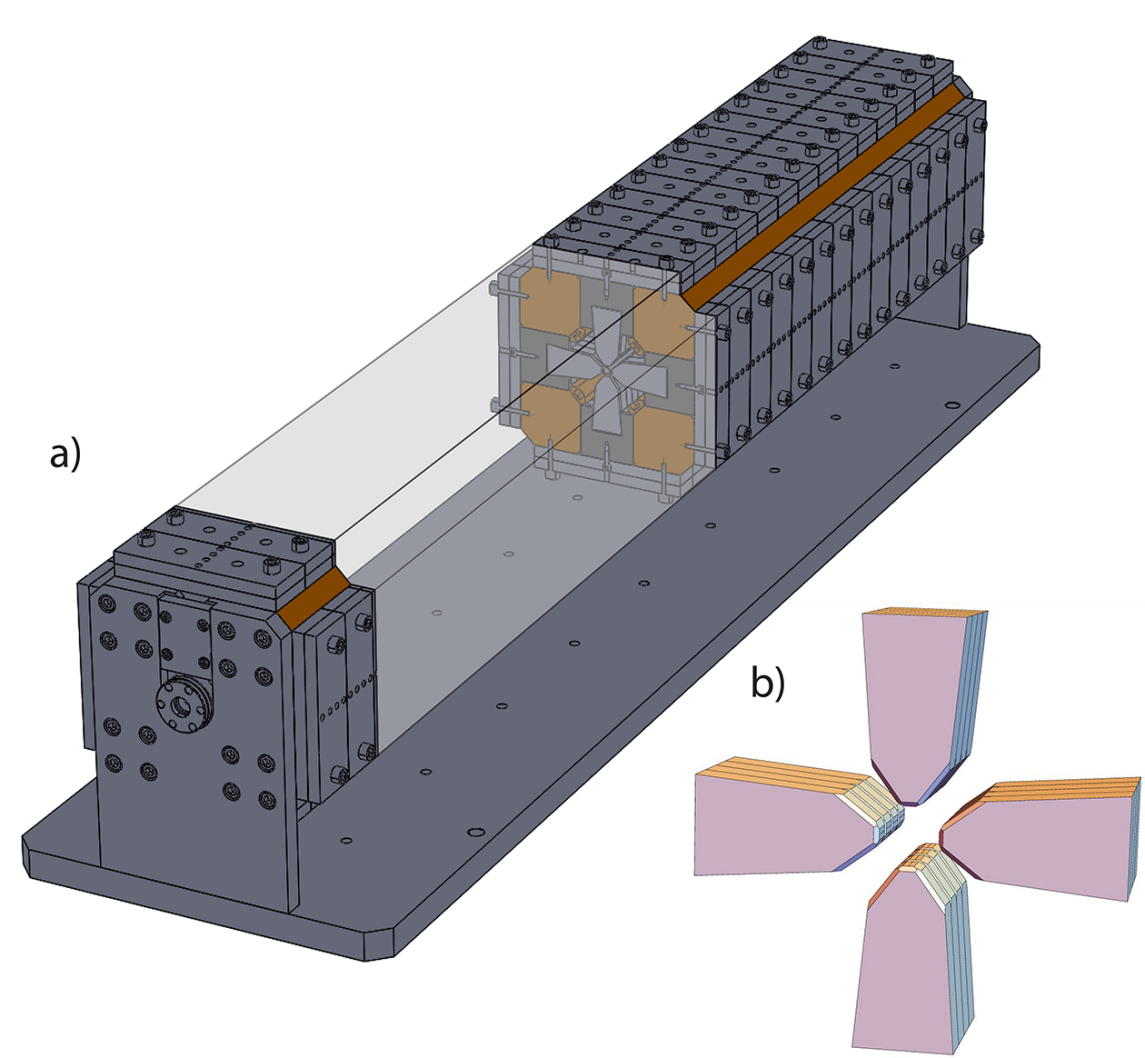
Theseus undulator. (*a*) Helical geometry consisting of two permanent-magnet Halbach arrays. (*b*) A single period modeled in *RADIA* with magnet chamfering.

The paper is organized as follows. We first present the most general expression for the field in a helical undulator and discuss the resulting beam dynamics, showing the importance of the off-axis fields in the optical properties of the device. We then discuss the pulsed-wire measurements for the Theseus undulators (Fig. 1 ▸) which utilize transverse wire scans to identify the magnetic axis as well as measure higher order field moments. In order to guide the tuning of quadrupole and sextupole components in the undulator field, we present an analytic model that accurately predicts the effect of varying magnet positions to correct the 3D fields and minimize deviations in the off-axis trajectories.

## 2. General field expansion and beam transport

The most general expression for the field of a helical undulator can be constructed from the separated variable solution to the Laplace equation for the magnetic scalar potential of a planar undulator array with λ_u_ = 2π/*k*_u_ periodicity in the *z*-direction. Superposing the potentials for two planar arrays, phase shifted by 90° and rotated with respect to each other, we can write 
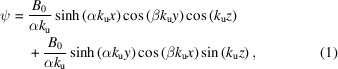
using normalized transverse wavenumbers α = *k*_*x*_/*k*_u_ and β = *k*_*y*_/*k*_u_, which satisfy α^2^ − β^2^ = 1 from the Laplace condition 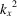 − 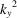 = 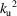.

**Figure 2:**
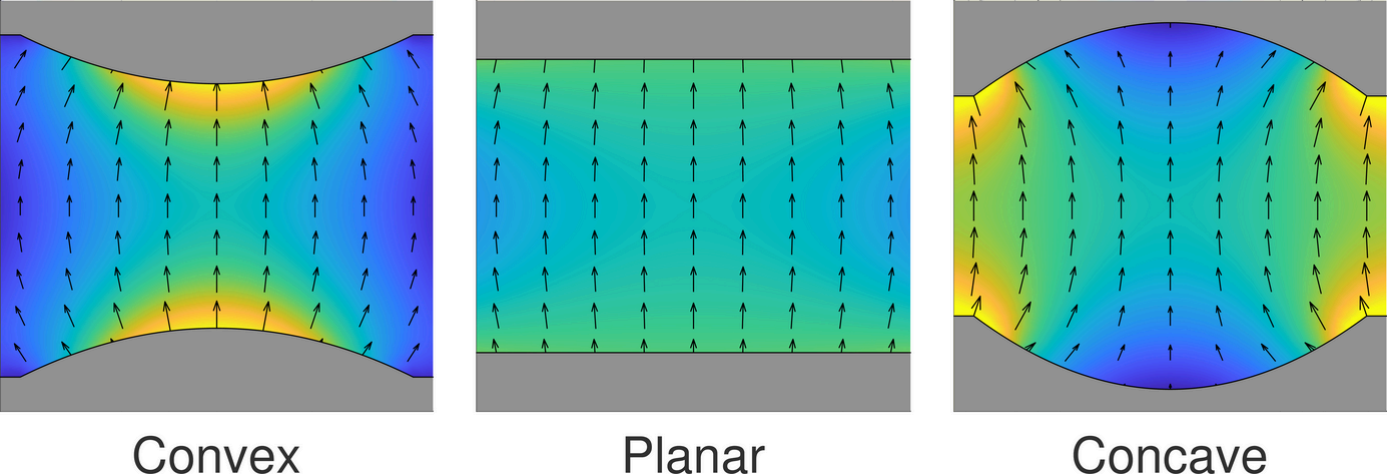
Effect of pole shaping on transverse magnetic fields in planar array.

In general, α is real due to the longitudinal fringe fields of each magnet while β can be real or imaginary depending on the magnetic profile due to the transverse pole geometry as shown in Fig. 2 ▸. For a concave pole shaping, the imaginary β results in an exchange between harmonic functions and hyperbolic functions. As an example, for the bifilar coil case of Madey’s original FEL we have α^2^ = 1/4 and β^2^ = −3/4 such that both traverse functions are hyperbolic. On the other hand, the Theseus undulators (Fig. 1 ▸) are designed with convex pole shaping to maximize the undulator field.

The magnetic field **B** = −∇ψ is the gradient of the scalar potential and an expression for the fields of the helical undulator near its axis can be obtained through a small-argument expansion for the hyperbolic and harmonic functions, 
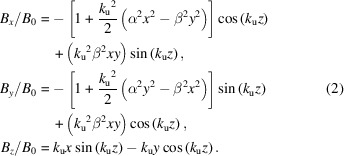
Now we review the dynamics of relativistic electrons in the helical undulator to highlight the role of α and β in the beam transport and the coupling between the transverse planes at low energy. Closely following the work of Ciocci *et al.* (1992 ▸) and Reiche (2018 ▸), it is helpful to start with the single particle Hamiltonian in terms of the undulator magnetic vector potential, 
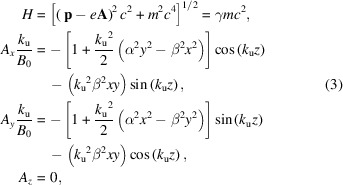
where the constant particle energy is defined in terms of the relativistic beam factor γ.

It is customary to evaluate the beam dynamics specified by the Hamiltonian equations of motion by separating the beam trajectory into a fast oscillation (**r**_f_ = 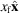 + 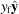) and slow drift (**r**_s_ = 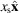 + 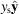). The fast oscillations are obtained by neglecting the off-axis contribution to the vector potential. In this case, we find the helical trajectory given by 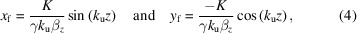
where *K* = *eB*_0_/*m*_e_*ck*_u_ is the undulator strength parameter. The longitudinal velocity associated with this motion is constant along the undulator,
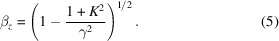
Substituting **r** = **r**_s_ + **r**_f_ and converting to spatial derivatives, the time-averaged equations of motion are given by 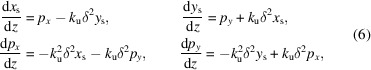
where δ = 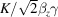 describes the orders of the relativistic approximation γ ≫ *K* and momentum has been normalized by γ*mc*β_*z*_. This system is notably independent of the magnet pole shapes and transverse wavenumbers since each nonvanishing term only contains the combination α^2^ − β^2^ which is equal to 1. In other words, though the off-axis field expansion can vary locally, the average effect on the particle trajectory depends only on the Laplace condition and is the same regardless of the details of the undulator magnet technology.

The system of coupled equations can be reduced to 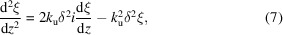
keeping the lowest order terms in δ where ξ = *x*_s_ + *iy*_s_. A guess of the form ξ = 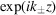 yields *k*_±_ ≃ *k*_0_(±1 + δ) to first order where *k*_0_ = 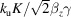 and the full solution is the linear combination ξ = 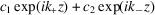 with complex constants. Expressions for *x*_s_ and *y*_s_ are found by taking the real and imaginary parts yielding 
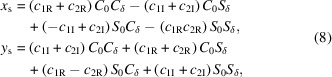
where we use the short-hand notation *S*_0_ = 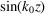, *C*_0_ = 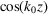, *S*_δ_ = 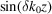 and *C*_δ_ = 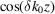. The real constants are expressed from the input beam coordinates as 
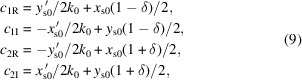
where 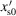 = d*x*_*s*0_/d*z* and 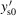 = d*y*_*s*0_/d*z* refer to the initial trajectory angles.

Recasting *x*_s_, 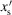, *y*_s_, 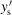 in terms of *x*_*s*0_, *y*_*s*0_, 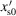, 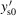, we can identify the 4 × 4 linear transfer matrix for the undulator as 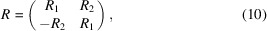
where 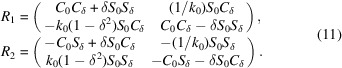
In the ultrarelativistic limit, δ → 0 and the *R*_1_ matrix provides the natural undulator focusing effect with the well known β = 1/*k*_0_ = 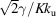. At lower beam energies the dynamics are more complex and one has to take into account the coupling terms. The *R*_2_ block matrix clearly indicates that low energy transport inside the undulator cannot be decoupled even in the linear transport approximation.

**Figure 3:**
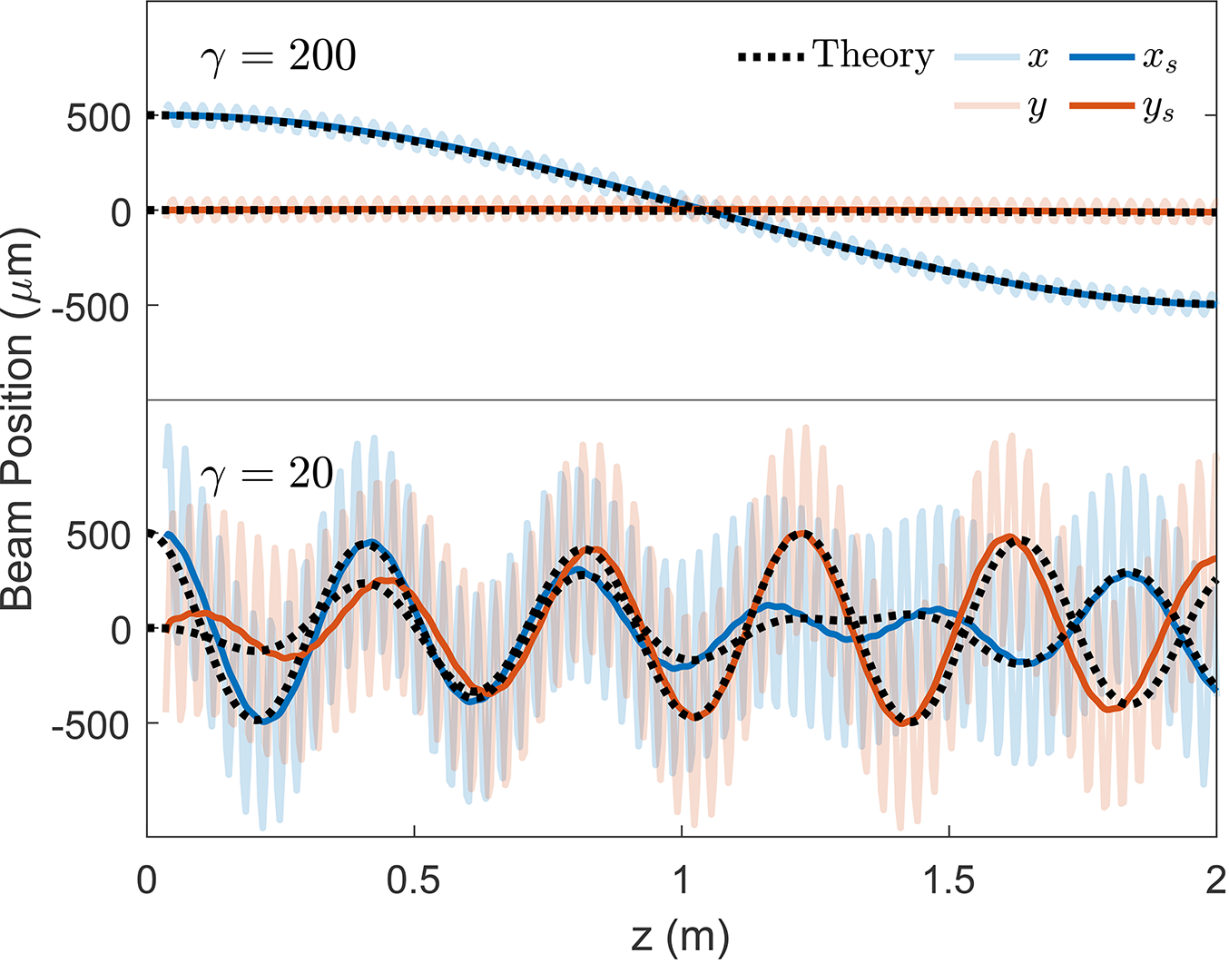
Comparing undulator focusing matrix transport against particle-tracking simulation at high and low beam energies. The period-averaged and full simulated trajectory (**r**_s_ and **r**) are plotted against the matrix transport theory.

We compare the linear matrix transport against numerical integration of the Lorentz force equation in Fig. 3 ▸ using the particle tracking code *GPT* (De Loos & Van Der Geer, 1996 ▸) at two different energies (relativistic factor γ = 200 and γ = 20) where the beams are given an initial *x*-offset of 500 µm in an undulator with *K* = 2.18. The period-averaged slow drift **r**_s_ is plotted on top of the semi-transparent trajectory **r**. At lower beam energies, significant coupling is introduced in addition to the shortening of the betatron oscillation period, but the matrix transport still accurately tracks the average position of the beam. Note that the angle of electron trajectories (due to either initial beam offset or increased geometric emittance) varies inversely with energy such that there is no additional redshifting of the emitted radiation at lower energies.

**Figure 4:**
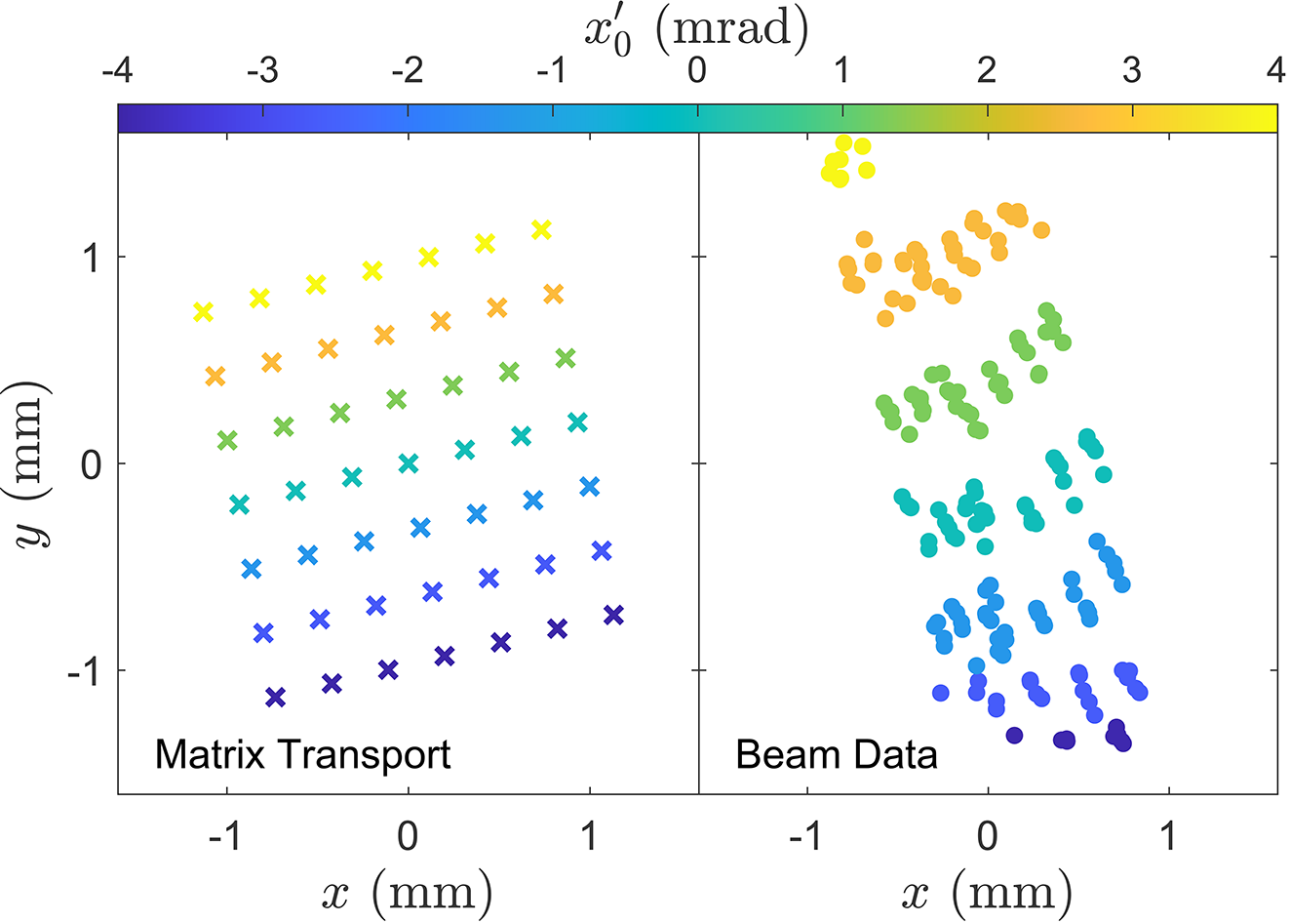
Comparison between matrix transport and beam measurement. A 2D raster scan of an upstream steering magnet (8 cm before undulator) varies the beam injection angle, and the centroid is measured on a screen 14 cm downstream of the undulator exit.

Fig. 4 ▸ compares the results of matrix transport through the Tessatron undulator against beam centroid measurements performed at the UCLA Pegasus laboratory (Fisher *et al.*, 2022 ▸) for an input beam energy of γ = 14.5. The beam centroid is measured on a screen 14 cm downstream of the undulator while the horizontal and vertical beam angles are adjusted with a calibrated steering magnet 8 cm upstream of the entrance. The orientation of the centroid measurements with respect to steering is consistent with matrix transport where the coloring highlights transverse coupling as changes in horizontal steering lead to vertical deflections out of the undulator. The asymmetry seen in the beam data can be produced in *GPT* simulations by varying the initial alignment into the undulator.

**Figure 5:**
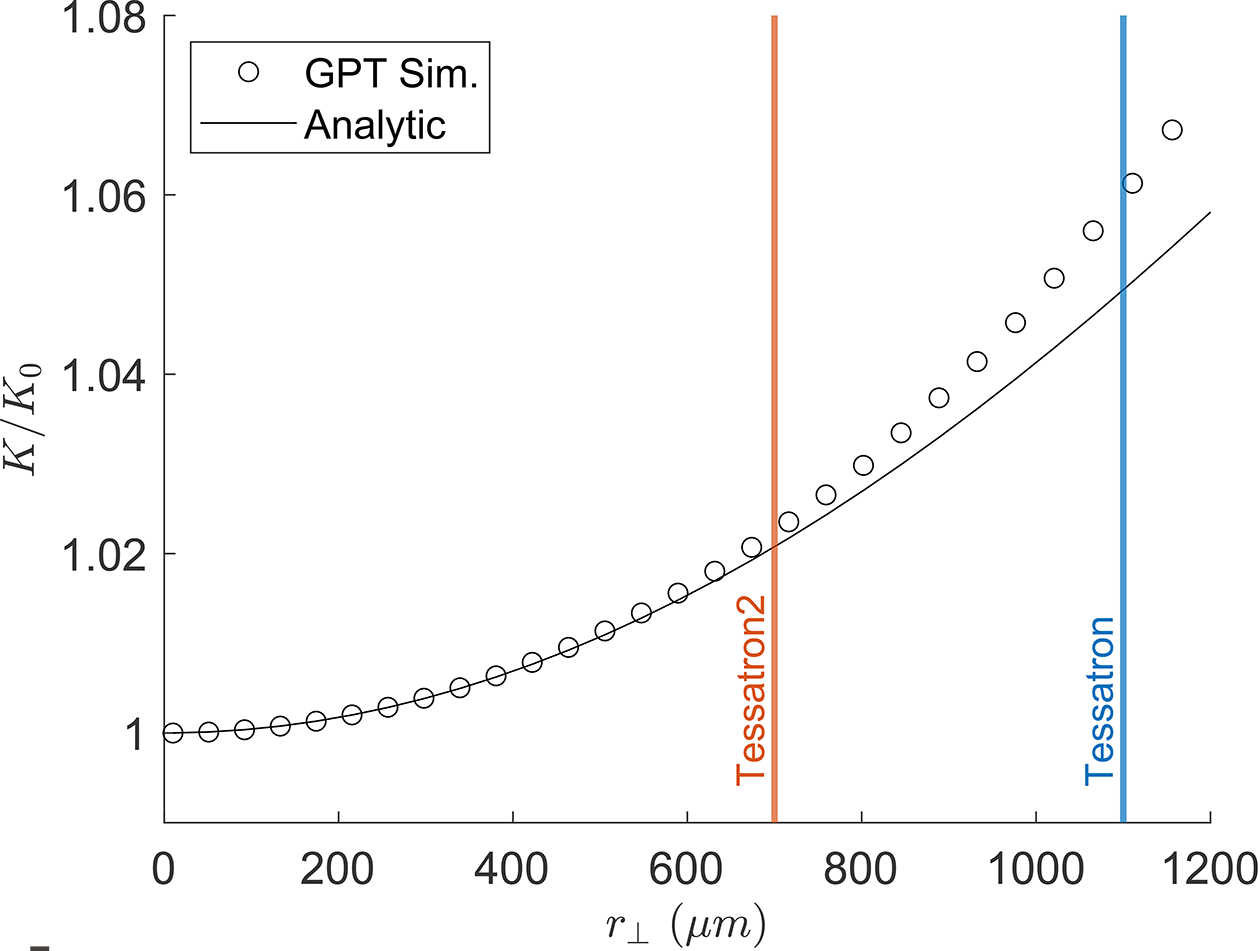
Corrections to the undulator strength parameter due to stronger transverse fields and non-zero *B*_*z*_ fields sampled off-axis.

In addition to coupled focusing, low energy beams have an increased wiggling amplitude such that the electrons sample stronger transverse fields and non-zero *B*_*z*_ fields that contribute to the Lorentz force and alter the undulator strength parameter defined by equation (5) which can alternatively be expressed as *K* = γβ_⊥_. The transverse velocity can be computed by considering the cyclotron motion in the transverse plane given by 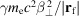 = |**F**_r_| with 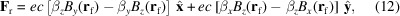
where the full radial Lorentz force is computed along the fast trajectory of equation (4) with the nominal parameter *K*_0_ = *eB*_0_/*m*_e_*ck*_u_. Using the fields from equation (3) and expanding to lowest order in *K*_0_ and γ gives 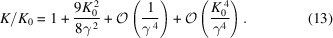
The strong focusing affects the transverse coupling and was included in the matrix transport of Figs. 3 ▸ and 4 ▸. Fig. 5 ▸ benchmarks equation (13) against *GPT* simulations as a function of wiggling amplitude using nominal parameters from the Tessatron THz-FEL experiments conducted at UCLA where simulated undulator parameters were computed from the average longitudinal velocity and γ factor of the beam. In this extreme example, operation at the zero-slippage waveguide resonance for the Tessatron experiments requires beam trajectories with an amplitude of roughly half the waveguide radius of 2 mm. There is little effect on resonance though, as the beam energy can be changed to compensate for errors in *K*.

## 3. Theseus pulsed-wire tuning

The large trajectory amplitudes at low energy present a challenge for undulator tuning as traditional Hall probe and pulsed-wire techniques focus mainly on tuning the on-axis fields. If not already prevented by the undulator geometry, 3D Hall probe scans would be time-consuming. Instead, we focus on generalizing pulsed-wire measurements to measure and tune the off-axis trajectories.

After a description of the pulsed-wire measurement bench, we will discuss the tuning procedures developed during commissioning of the Theseus undulators [described extensively by Park *et al.* (2017 ▸) with parameters reported in Table 1 ▸]. The magnetic field, designed with the 3D magnetostatic simulation code *RADIA* (Chubar *et al.*, 1998 ▸), is fit to equation (3) with the parameters α = 1.534 and β = 1.163. This implicitly assumes an ideal sinusoidal longitudinal dependence, but it was verified that higher order harmonics were negligible. The full undulator periods are initially tuned to minimize the phase error between the beam and radiation by pulling a three-axis Hall probe along the undulator with a 1 m translation stage as described by Park *et al.* (2022 ▸) with corrections for errors in probe position and angle.

### 3.1. Setup

**Figure 6:**
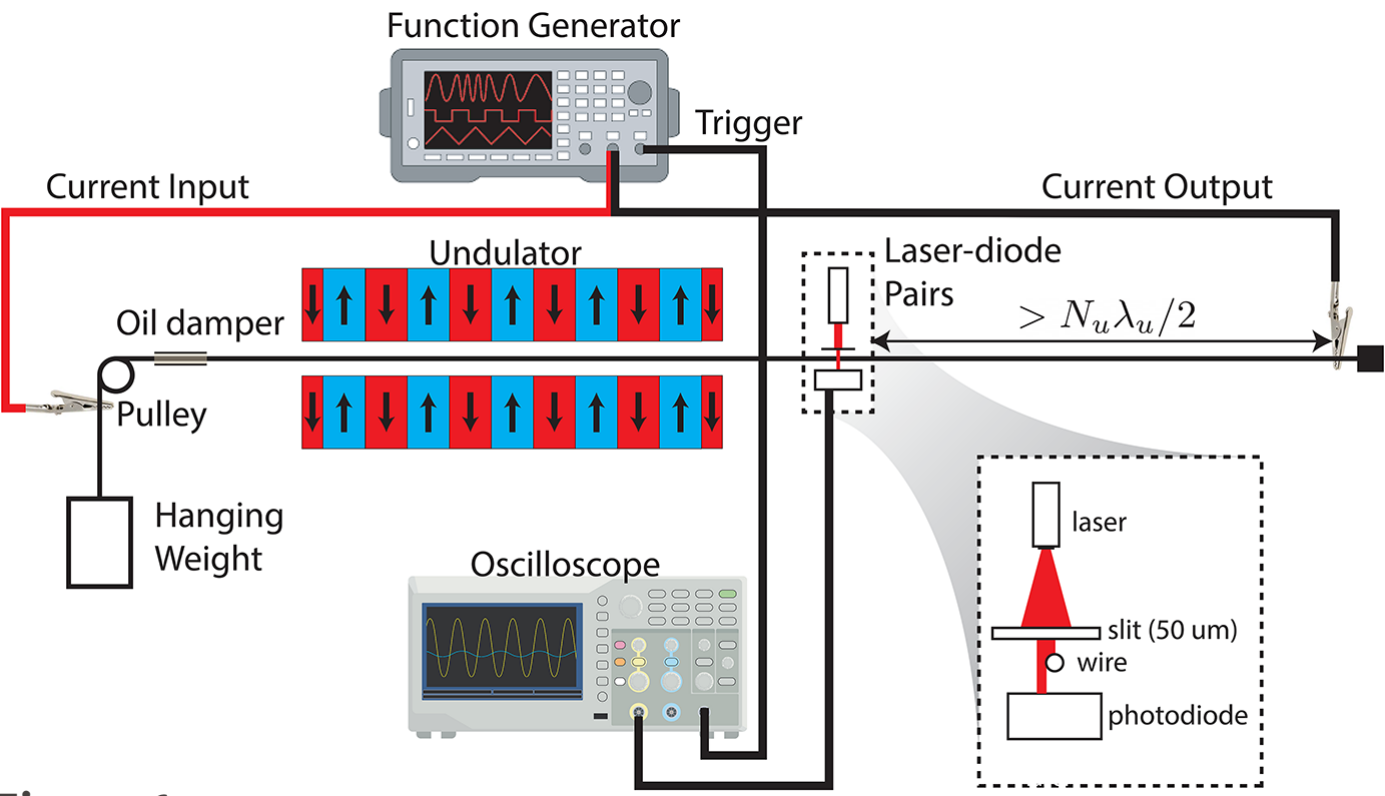
Schematic of the the pulsed-wire measurement bench.

The pulsed-wire technique allows instantaneous measurements of the undulator field integrals along the wire axis (Warren, 1988 ▸; D’Audney *et al.*, 2016 ▸). Fig. 6 ▸ shows a schematic of the measurement bench where a 50 µm-diameter CuBe wire is strung through the undulator and tensioned over a pulley with a hanging weight. A function generator passes square current pulses through the wire such that the magnetic forces excite a pair of left/right traveling waves. For an undulator with *N*_u_ periods of length λ_u_, the time-dependent wire deflection can be shown proportional to the first or second field integral in the limit of short (Δ*t*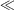 λ_u_/*v*_0_) or infinitely long (Δ*t* > *N*_u_λ_u_/*v*_0_) pulse durations, 
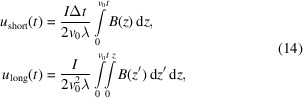
where *I* is the pulse current, λ is the linear mass density of the wire, and *v*_0_ is the wave velocity.

The wire deflection is measured in both transverse dimensions with laser–photodiode pairs. The inlay shows that after passing through a 50 µm slit the laser light is partially blocked by the wire such that the photodiode voltage varies in relation to the wire displacement. Nonlinearity is introduced due to diffraction between the slit and wire. To allow a full undulator measurement before interference from reflections, the wire must extend at least half the undulator length beyond the laser–diode pairs. Measurements are captured on an oscilloscope triggered by the function generator. Oil dampers enable higher repetition rates such that errors can be reduced by averaging over many (∼30) shots, while also damping noise due to air currents and table vibrations.

**Figure 7:**
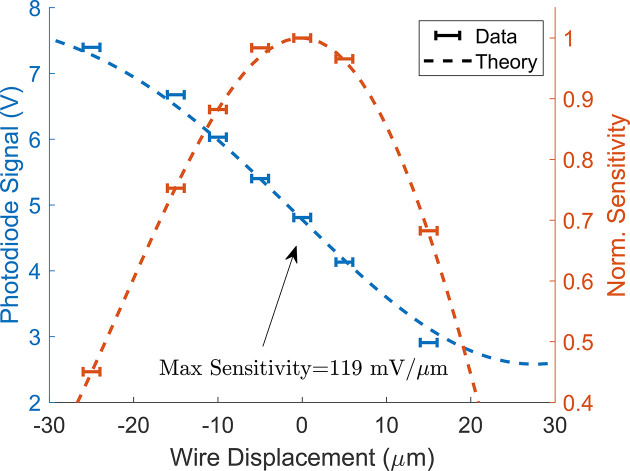
Pulsed-wire calibration. Sensitivity is defined as change in voltage over wire displacement. Normalized sensitivity is proportional to sinusoidal amplitudes of trajectory measurements.

A calibration of the photodiode voltage is shown in Fig. 7 ▸ as a function of wire displacement in good agreement with numerical simulations including diffraction. The nonlinear dependence is easier to observe in the measurement sensitivity, defined by the derivative of the voltage with respect to the wire displacement. Notably, the normalized sensitivity is proportional to the change in trajectory amplitude as the laser–photodiode pair is moved across the wire. Our setup achieves a maximum sensitivity of 119 mV µm^−1^ and, though resolution was limited by the 8-bit oscilloscope, a higher sensitivity improves the signal to noise ratio.

Common limitations to the ideal pulsed-wire theory in equation (14) include wire sag and dispersion. The sag *s*(*z*) can be modeled as a catenary in terms of the linear mass density, tension *T*, and wire length *L* by 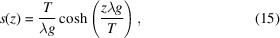
where *g* = 9.81 m s^−1^ and the ratio *T*/λ = 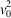 is notably independent of the wire diameter. The maximum sag can be approximated by *s*(*L*/2) − *s*(0) ≃ *g*λ*L*^2^/8*T*. For our measurements using 2 m of CuBe wire with 8.25 g mm^−3^ density and 1000 MPa tensile strength, the 50 µm sag is negligible. For longer undulators, ultralight dielectric threads have been considered for supporting the wire, but it is often simpler and sufficient to account for the sag through a numerical correction to the field integral (Varfolomeev *et al.*, 1995 ▸; Osmanov *et al.*, 1998 ▸).

**Figure 8:**
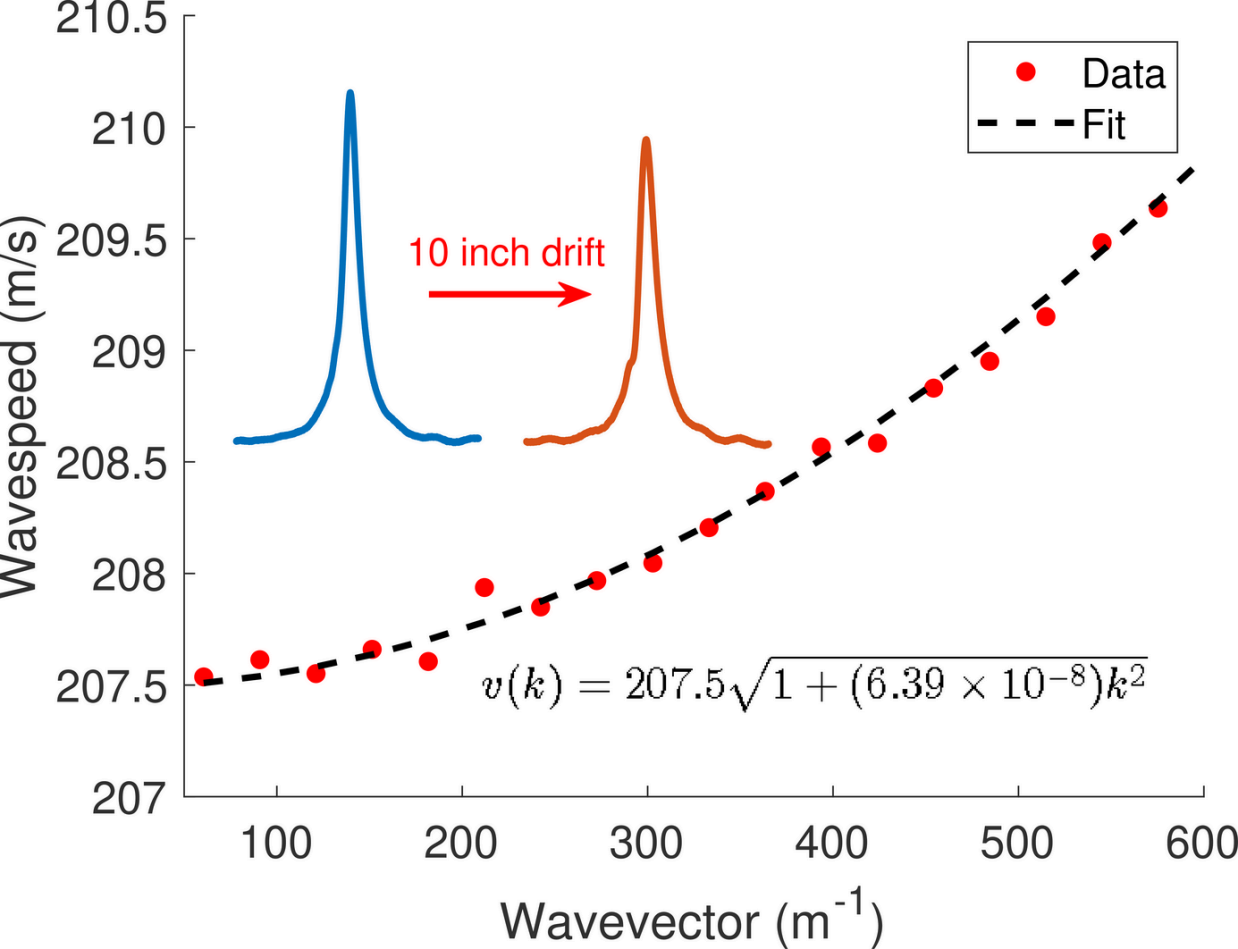
Pulsed-wire dispersion fit to Euler–Bernoulli theory from two laser measurements with 10-inch separation. Using the dispersion fit, distorted measurements can be corrected with numerical algorithms.

The dispersive wave velocities can be derived using a Euler–Bernoulli analysis of beam deflection as *v*(*k*) =*v*_0_(1 + *k*^2^*EI*/*T*)^1/2^ where *EI* is the flexural rigidity and *v*_0_ is the velocity in the low-frequency limit. These dispersion parameters can be inferred from two measurements of a large-bandwidth pulse by fitting the phase advance of the Fourier components, Δϕ = −ωΔ*z*/*v*(*k*), as shown in Fig. 8 ▸. With known dispersion, the effects on the measured signal can be removed using appropriate correction algorithms (Arbelaez *et al.*, 2013 ▸; Kasa, 2018 ▸). Distortion is largely dependent on the bandwidth of the measurement with the strongest effects seen at the beginning and end of the signal. By maximizing the wire tension, we achieved a relatively small parameter, *EI*/*T* ≃ 6.4 × 10^−8^ m^2^, such that the dispersion in the 1 m undulator for our narrow-bandwidth trajectory had negligible effect on wire alignment and trajectory tuning.

### 3.2. Wire alignment

**Figure 9:**
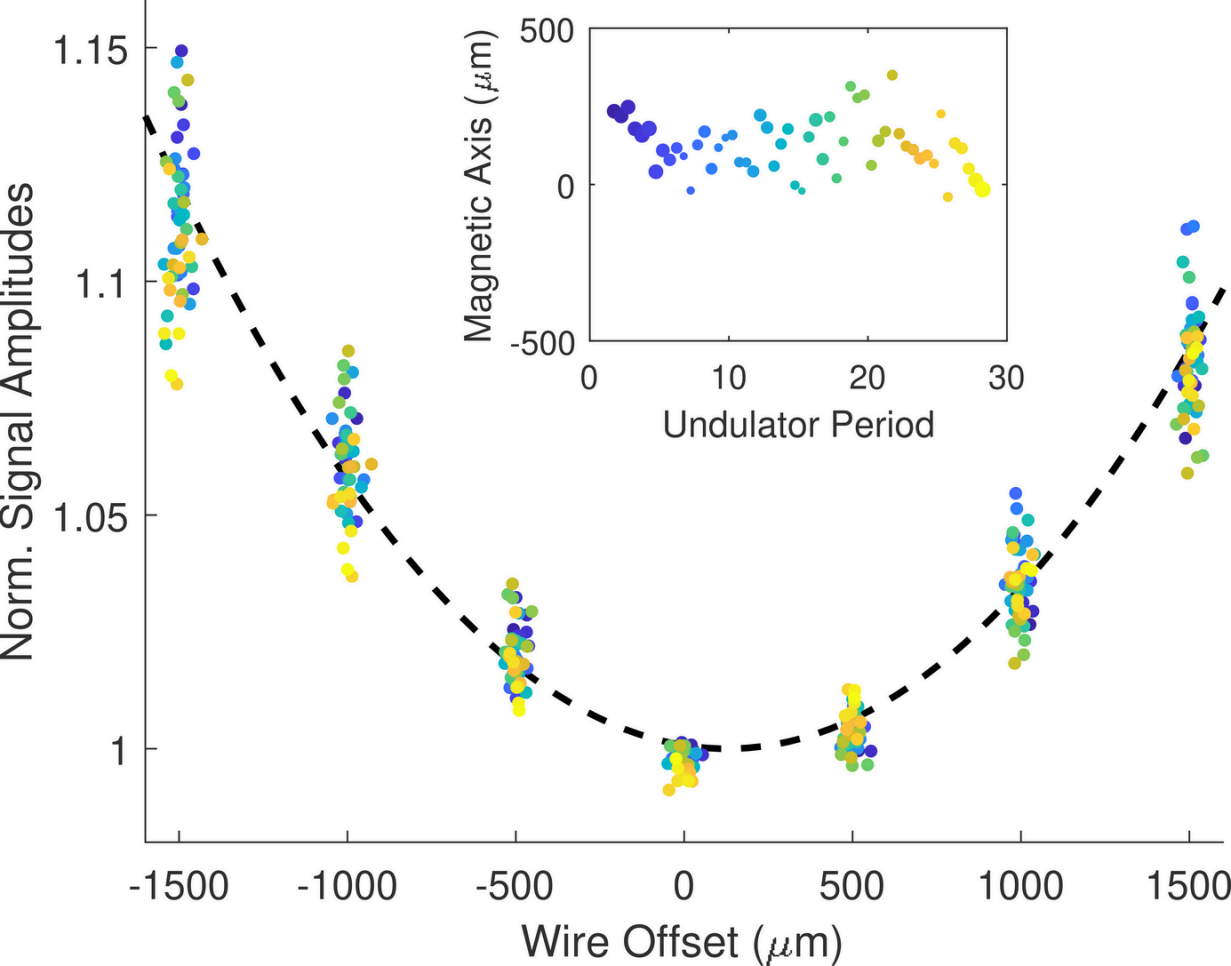
Wire alignment. The position of the magnetic axis along the undulator is inferred from the variation in field strength given by the change in signal amplitudes. The inlay shows the symmetry axes of quadratic fits at each magnet position along the undulator.

Before tuning, the wire must be aligned to the undulator magnetic axis. The tensioned wire behaves like a high energy (>1 GeV) beam that is little affected by undulator focusing such that the relative field strengths can be inferred from trajectory amplitudes at different transverse wire positions. Quadratic fits of the field concavity at each magnet provide an estimate of the magnetic axis along the undulator. Fig. 9 ▸ shows data for normalized amplitudes in 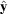 as a function of wire offset in 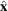, matching the theoretical concavity 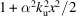 denoted by the black curve. The inlay plots the symmetry axes of the quadratic fits with marker size determined by the fit residuals, allowing repeatable identification of the magnetic axis to an accuracy of 100 µm.

The concavity fits are better constrained with large wire offsets, but field errors can produce strong deflections in the wire such that the sensitivity changes appreciably along the measurement (see Fig. 7 ▸). This is corrected with a non-linear calibration of the normalized sensitivity as a function of voltage *S*(*v*) through repeated measurements where the laser is scanned across the wire. Additionally, strong linear deflections of the sinusoidal waveform shifts peak positions such that measured peak-to-peak amplitudes are reduced. From a simple analytic model, it can be shown that the peak-to-peak amplitude is decreased by a factor 1 − (1/2)(Δ/2π)^2^ where Δ is the change in voltage over a period divided by the sinusoidal amplitude.

### 3.3. Higher-moment tuning

**Figure 10:**
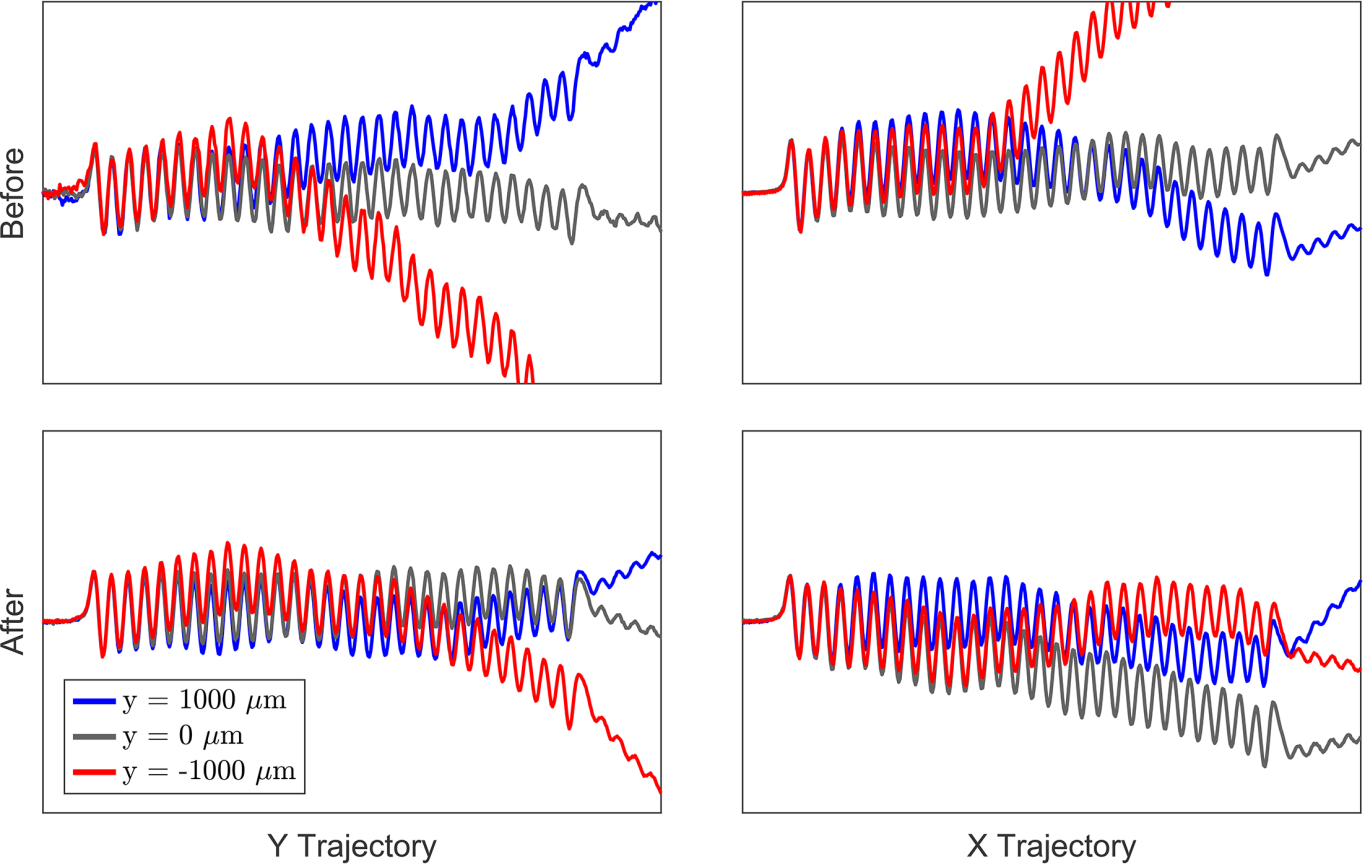
Off-axis pulsed-wire measurements before and after 3D pulsed-wire tuning.

By measuring local field concavity, we can identify the magnetic axis along the undulator. However, it is also necessary to minimize errors in the beam trajectory as given by the dynamic integral over the sampled off-axis fields. In fact, it is good to remember that in a helical trajectory the beam never sees the on-axis fields. The enclosed undulator geometry restricts Hall probe mapping of the 3D fields, but static pulsed-wire measurements can still be used to identify and tune higher-order field moments. For example, Fig. 10 ▸ shows pulsed-wire measurements of the beam trajectory after Hall probe tuning for the Tessatron THz-FEL experiment, before and after 3D pulsed-wire tuning. Ideally, the measured trajectories would be straight and independent of the wire’s transverse position, but the initial off-axis measurements clearly exhibit strong deflections at localized magnet positions corresponding to quadrupole and sextupole errors. Without further tuning, these moments would introduce errors in the trajectories of low energy beams which, though limited by strong undulator focusing, could result in diminished charge transmission and FEL performance due to the tight alignment tolerances required near the zero-slippage condition. Beam-based tuning methods could not provide local tuning information and would be confused by the strong undulator focusing.

To describe the effect of magnet adjustments on undulator fields and integrated beam trajectories, we examine a simple empirical toy-model where a pair of undulator magnets is represented by two pure magnetic dipoles. The strength and separation of the dipoles are fit to *RADIA* simulations of Theseus magnets with *B*_0_ = 730 mT peak field and 7.1 mm full-gap such that each magnet is best modeled by a dipole with *m* = 1.59 × 10^6^ A mm^2^ located 7.33 mm inside the magnet tip. While this model is fit to fields, we later verify that it performs sufficiently well in describing the effect of tuning on beam trajectory.

**Figure 11:**
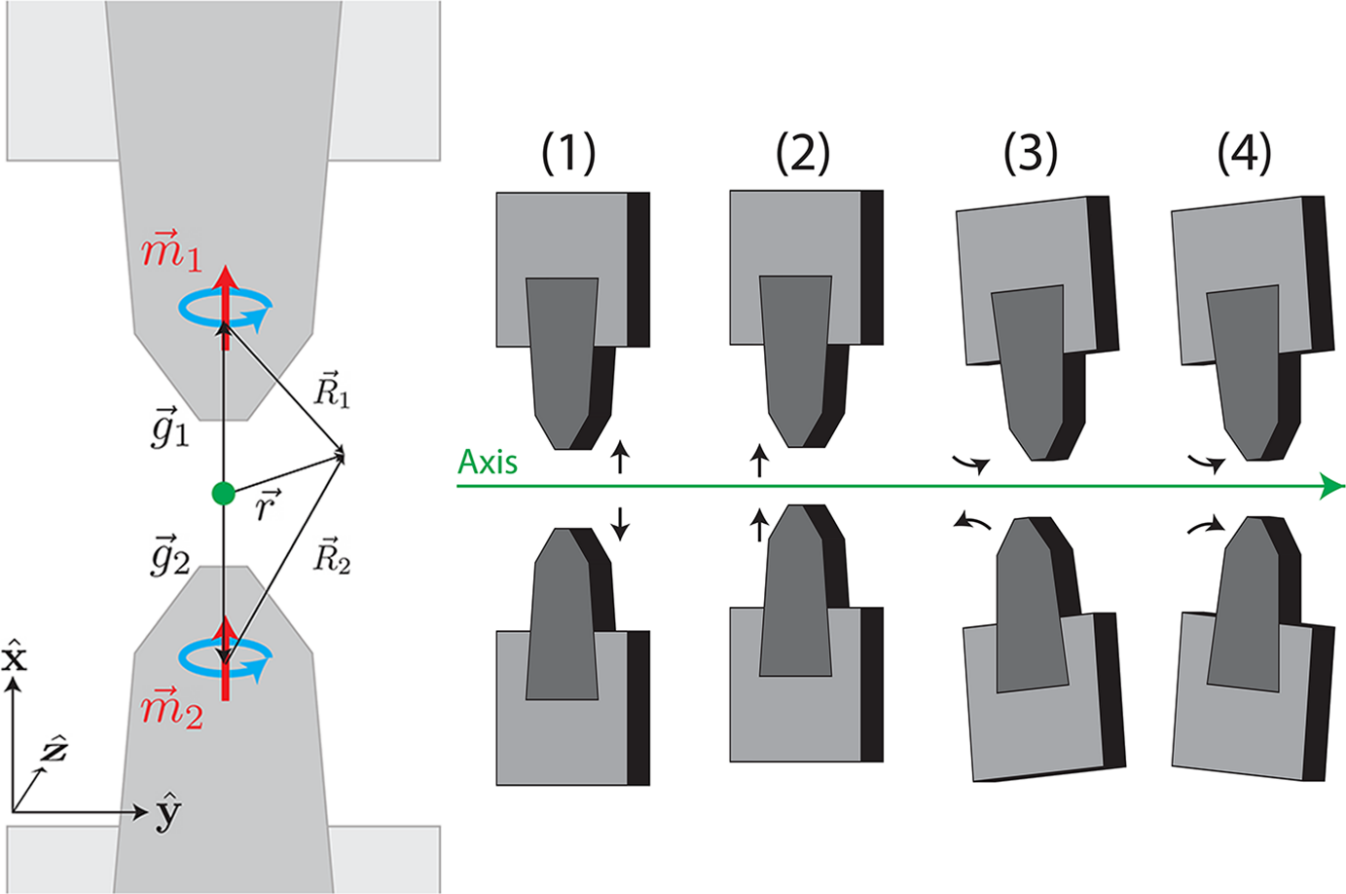
Schematic of toy dipole model. The four tuning cases represent changes in magnet gap and magnetization angle in the transverse plane.

In our model, dipoles are nominally placed at **g** = 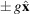 with moments **m** = 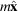 as shown in Fig. 11 ▸. We allow small adjustments to the position (δ*g* in 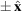) and magnetization angle (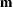 = 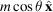 + 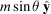) to simulate tuning of the magnet gaps and slight changes in the magnet angle, where the tuning space is spanned by four different cases.

The fields of each dipole are expressed by 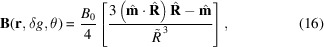
where *B*_0_ = *m*μ_0_/π*g*^3^ and 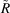 = *R*/*g* where tildes generally indicate normalization by the nominal half-gap *g*. Summing both dipole fields and Taylor expanding about the axis yields the dipole field with a higher-order skew sextupole field as expected from the general undulator expansion in equation (3),
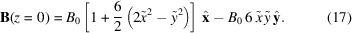
Note that the asymmetry of the dipole model (α/β = 1.414) is in fairly good agreement with the Theseus fields (α/β = 1.319). It is also clear that the strength of the higher-order moments depends on the ratio of the electron beam trajectory to the magnetic gap. The transverse velocity is given by an integral over the Lorentz force as 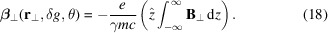
Remembering 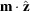 = 0, the integral over the transverse field can be evaluated in terms of **R**_⊥_ for each dipole as 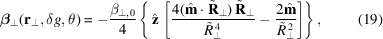
where β_⊥,0_ = *egB*_0_/γ*mc*. To describe the net change in beam trajectory, we consider the relative change in the transverse velocity given by 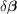 = 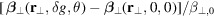. Summing the contributions of each dipole for the four cases and expanding to lowest order yields 
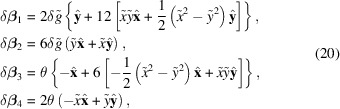
where 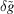 = δ*g*/*g* and terms are grouped into different magnetic moments. The first and third cases show that altering the magnet position or angle in opposite directions can be used to tune the dipole fields in 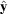 and 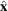. To simultaneously tune the regular and skew sextupole moments, one must utilize adjustments in the other magnet array with magnetization in 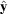 found by the coordinate transformation *x* → *y* and *y* → −*x*. Quadrupole fields can be tuned using the second and fourth cases where the magnet position or angle are altered similarly and only skew or regular quadrupole moments are generated to first order.

**Figure 12:**
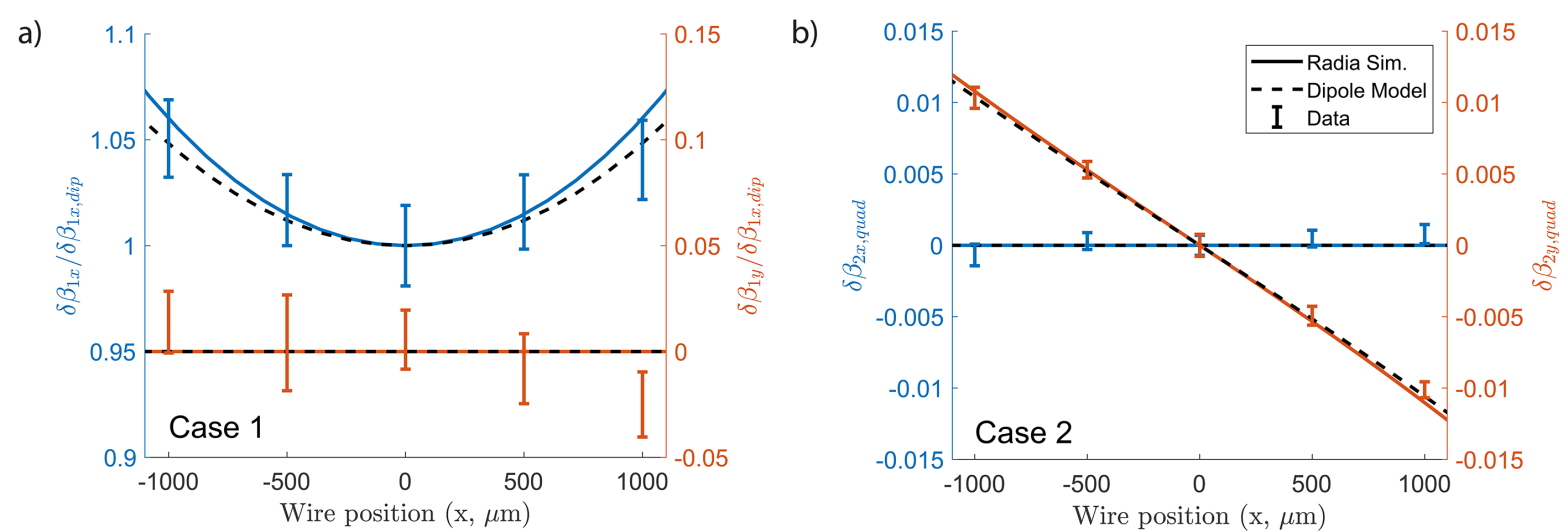
A comparison of measured and theoretical deflections due to magnet tuning as a function of transverse wire position. For case 1, opening the magnet gaps leads to a clear sextupole dependence on top of the dipole kick. In case 2, shifting the magnet gap creates a quadrupole dependence. Measurements are in agreement with *RADIA* simulations and the dipole model.

Fig. 12 ▸ benchmarks the analytic dipole model against pulsed-wire data and an ideal *RADIA* simulation using numerical integration of Theseus magnet fields. The first two cases are considered as it is easier to accurately tune the magnet gaps with fine adjustments. Both components of 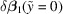 and 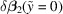 are plotted as a function of wire position along the *x*-axis. The magnet gaps are tuned by δ*g* = 200 µm which corresponds to a quarter-turn of the 8–32 tuning screws with 784 µm rev^−1^ pitch.

Pulsed-wire measurements of the beam trajectory were taken in both transverse dimensions at each wire position before and after the tuning of a magnet pair. For each offset, reference measurements are subtracted and the residual signals are period-averaged to eliminate any remaining periodicity. Linear fits are applied to the waveform before and after the tuned magnet pair such that the change in slope is proportional to the change in velocity. The proportionality constant includes the calibrated conversions from time and voltage to position, *i.e.* the voltage amplitude of an individual measurement can be related to the nominal beam trajectory amplitude in the undulator. The data are plotted with error bars showing the standard deviation of 30 measurements.

Fig. 12 ▸(*a*) shows the net change in normalized velocity of the first case normalized by the dipole field. The measurement variation is relatively large as the concavity of the sextupole moment occurs on top of a large dipole field. This produces a strong deflection in the wire requiring corrections to the non-uniform sensitivity along the measurement and reducing the voltage resolution of the 8-bit scope measurement. The data show a clear sextupole dependence in 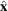 with minimal effect in 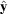. In the figure we also show the results of *RADIA* simulations where the same magnet adjustments are performed on the Halbach array. The agreement between the dipole model, the simulations and the data is remarkable and can be used to guide the undulator tuning with quick convergence. In Fig. 12 ▸(*b*) we show the quadrupole moments resulting from shifting the magnet offsets in the same direction (*i.e.* second case from Fig. 11 ▸). In this case, the measurements, which match closely the prediction from our simple two-dipole model and the *RADIA* simulations, are more accurate since there is no undulator dipole component and the variance is smaller.

Guided by equation (20), appropriate magnet pairs can be adjusted to tune the deflections seen in Fig. 10 ▸. In practice, the corrections are limited by the tuning range and precision when adjusting magnet angles. The UCLA THz-FEL system is particularly robust to phase errors between the short beam and radiation (10%) as the rapid field growth produces a deep ponderomotive potential well where the particles are stably trapped. The phase error after Hall probe tuning was only 1% such that the main limitation for high efficiency is charge transmission through the undulator. The red and blue trajectories in Fig. 10 ▸ measure the field integrals along the wire axis and do not exactly represent the fields seen by the helical trajectory of a low energy beam. Instead, we approximate the true beam trajectory as the average of pulsed-wire measurements at wire offsets of ±1000 µm in 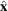 and 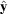. The final averaged trajectories are tuned flat to a centroid deviation of less than 300 µm from the axis, sufficiently smaller than the millimeter-scale wiggling amplitude and the 2 mm waveguide inner radius employed in the experiments.

## 4. Conclusion

The Tessatron experiment uses zero-slippage operation in a helical undulator to demonstrate high energy extraction from a relativistic beam. However, the enclosed geometry and low beam energy complicates undulator tuning and beam dynamics. Starting from a general description of helical undulator fields, we derived the linear matrix transport describing coupling in the betatron focusing between the transverse planes and compared with particle-tracking simulations and experimental data.

In order to optimize the particle trajectory and transport at these low energies we implemented an upgraded pulsed-wire measurement bench at UCLA where it is possible to measure the beam trajectory at various transverse wire positions to infer the magnetic axis from the local field concavities. A toy-model was developed to understand how changing position and angles of opposite magnet pairs in the arrays can be used to correct the higher order undulator field components and tune the off-axis trajectories. The model was validated against numerical simulation and pulse-wire measurements and eventually used to optimize the trajectories before successful deployment of the insertion device on the UCLA Pegasus beamline. A number of experiments at various injection energies were carried out using the undulator tuned as described in this paper (Fisher *et al.*, 2022 ▸; Lenz *et al.*, 2022 ▸). It is worth noting here that in our setup the 3D pulse-wire-based tuning was ultimately limited by the range and accuracy of magnet angle adjustments. Additionally, it did not account for longitudinal magnetization errors or tuning of the longitudinal magnets in the Halbach array. These shortcomings should be corrected in future versions of permanent-magnet helical undulators to ensure optimal performances for the widest energy range.

## Figures and Tables

**Table 1:** Theseus undulator parameters

Period length	32 mm
Undulator length	28λ_u_
Magnetic field	0.67–0.83 T
Normalized vector potential, *K*	2–2.5
Magnetic gap	5.58–7.54 mm
Magnet type	NdFeb
Remnant field	1.18 T
